# Nano-biolistics: a method of biolistic transfection of cells and tissues using a gene gun with novel nanometer-sized projectiles

**DOI:** 10.1186/1472-6750-11-66

**Published:** 2011-06-10

**Authors:** John A O'Brien, Sarah CR Lummis

**Affiliations:** 1 Neurobiology Division, MRC Laboratory of Molecular Biology, Cambridge CB2 2QH, UK; 2 Department of Biochemistry, University of Cambridge, Cambridge CB2 1QW, UK

## Abstract

**Background:**

Biolistic transfection is proving an increasingly popular method of incorporating DNA or RNA into cells that are difficult to transfect using traditional methods. The technique routinely uses 'microparticles', which are ~1 μm diameter projectiles, fired into tissues using pressurised gas. These microparticles are efficient at delivering DNA into cells, but cannot efficiently transfect small cells and may cause significant tissue damage, thus limiting their potential usefulness. Here we describe the use of 40 nm diameter projectiles - nanoparticles - in biolistic transfections to determine if they are a suitable alternative to microparticles.

**Results:**

Examination of transfection efficiencies in HEK293 cells, using a range of conditions including different DNA concentrations and different preparation procedures, reveals similar behaviour of microparticles and nanoparticles. The use of nanoparticles, however, resulted in ~30% fewer damaged HEK293 cells following transfection. Biolistic transfection of mouse ear tissue revealed similar depth penetration for the two types of particles, and also showed that < 10% of nuclei were damaged in nanoparticle-transfected samples, compared to > 20% in microparticle-transfected samples. Visualising details of small cellular structures was also considerably enhanced when using nanoparticles.

**Conclusions:**

We conclude that nanoparticles are as efficient for biolistic transfection as microparticles, and are more appropriate for use in small cells, when examining cellular structures and/or where tissue damage is a problem.

## Background

Gene delivery using biolistics is a useful mechanism to transfect DNA into cells that cannot readily be transfected by other methods, and also has potential for delivery of other macromolecules such as RNA [1.2]. The technique was originally widely used for plant transfections [3], but, as it is a physical and not a chemical transfection procedure, its use is not limited to compliant cell types. It has, for example, been successfully used to transfect neurones (some of which are notoriously difficult to transfect), cells deep in tissues (DNA can be carried considerable distances through other cells such as layers of skin), and bacteria [4-7]. Currently there is much interest in its use for nucleic acid mediated immunizations; studies in the 1990s revealed that DNA vaccination could mediate protective immunity, as plasmid DNA incorporated into cells results in an antigen-specific antibody responses (see [8-10] for reviews).

A variety of systems have been developed for biolistic transfections and currently the Helios gene gun (Bio-Rad, Hercules, CA) is one of the most widely used. This gene gun, which delivers particles superficially over a relatively wide area, has proved useful for cultured cells or thin tissue sections, and the use of a modified barrel has allowed deeper, more targeted delivery [11]. Use of either barrel, however, results in tissue damage e.g. loss of cultured cells at the centre of the 'shot', or significant numbers of damaged cells in tissues [11-14]. This is problematic as the gene gun has considerable potential for use in human and animal gene therapy, and for transfecting delicate cell preparations. One potential route to decrease tissue damage is to decrease the size of the particles carrying the DNA. Most protocols currently use microparticles, which are usually ~1 μm diameter projectiles e.g. [1,2,11-15], but smaller particles (100-180 nm) have been used with some success [16,17]. The use of smaller particles has the potential advantage of allowing more efficient transfection of smaller cells and specific cellular regions. For example, one of us has recently been examining the structures of dendritic spines using diolistics (insertion of dyes into cells using projectiles) [18]. The length of these spines (0.5 - 3 µm) required the use of nanometer-sized projectiles, which have recently become available. However it was not clear if such particles would be suitable for carrying DNA into cells, and so in this study we have examined the efficiency, depth penetration and tissue damage of the 40 nm projectiles as compared to 1 μm projectiles.

## Methods

### Micro and nano-particles

**Figure 1:**
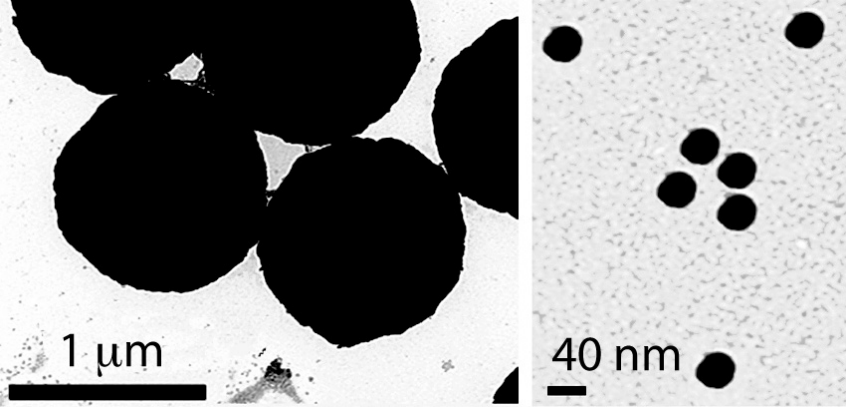
**Nanoparticles and microparticles**. Electron microscope images of microparticles (left hand panel; scale bar = 1 μm) and nanoparticles (right hand panel; scale bar = 40 nm) to show their comparative sizes. The particles in these images were coated with DNA and have hydrodynamic diameters of 40 and 991 nm respectively.

The core diameter of the particles used was 40 ± 0.8 nm or 991 ± 11 nm for the 40 nm (Alfa Aesar, MA, USA) and 1 μm (Bio-Rad laboratories, USA) particles respectively. These particles have a hydrodynamic diameter of 47.2 ± 1.3 and 1060.7 ± 2.4 nm respectively as determined by light scattering. Their electrokinetic properties are similar (54 mV and 55 mV respectively). To confirm the sizes we also examined the particles using electron microscopy (Figure 1).

### Preparation of projectiles

We define projectiles as gold particles upon which DNA has been precipitated by the use of spermidine and Ca^2+^. Electron microscope images (Figure 1) reveal that the size when coated is similar to uncoated particles i.e. 40 nm and 1 µm. Particles were prepared as previously described [15] using 1 µm or 40 nm diameter gold particles. Briefly, 50 µl of 0.05 M spermidine and 10 µl DNA at 0.5, 1, 2 or 4 mg/ml (pEYFP-N1; Clontech, USA) were added to 10 mg particles (final amounts of DNA/bullet were 0.25, 0.5, 1 or 2 μg). The mix was agitated by vortexing while adding 50 µl 1 M CaCl_2 _in 10-15 µl drops. After 5 min with intermittent mixing the supernatant was removed by centrifugation (1,000 × g for 30 s) and the gold pellet resuspended in 3.5 ml 0.075 M polyvinylpyrrollidone (PVP; Sigma). This suspension was then inserted into Tefzel tubing (0.1 mm internal diameter; Bio-Rad), the gold particles allowed to settle, and the supernatant removed. Then the tubing was rotated to ensure an even spread of the gold particles, which were subsequently dried with a flow of nitrogen. The tubing was cut using a tubing cutter (Bio-Rad) into 1 cm lengths to create bullets, which were either used immediately or stored with desiccant at 4°C until required.

### Biolistic transfection

Human embryonic kidney HEK293 cells were maintained in DMEM/F12 media at 7% CO_2_. For transfection they were grown on 22 mm diameter glass coverslips in 35 mm plates until 60-80% confluent. They were biolistically transfected with the modified gene gun [11] loaded with the coated projectiles using a gas pressure of 50 psi at a distance of 1 cm. After 24 h they were fixed with 4% paraformaldehyde (PFA; Sigma-Aldrich), counterstained with diamidino-2-phenylindole (DAPI; Vector), and mounted using Vectashield (Vector). Samples of adult mouse ear tissue (removed from dead mice) were shot with the modified gene gun using a gas pressure of 75 psi at a distance of 5 mm. They were fixed in 4% PFA and 50 µm sections obtained using a microtome. The sections were counterstained with DAPI for 5 min. Brain slices were prepared as previously described [11] and transfected with the modified gene gun using a gas pressure of 50 psi at a distance of 10 mm. After 24 h they were counterstained with DAPI for 5 min. No significant aggregation of particles was observed either before or after transfection. All images were viewed using a Bio-Rad Radiance Plus confocal microscope. Data is presented as mean ± SEM.

## Results

### Assessing the efficiency of nanoparticles

**Figure 2:**
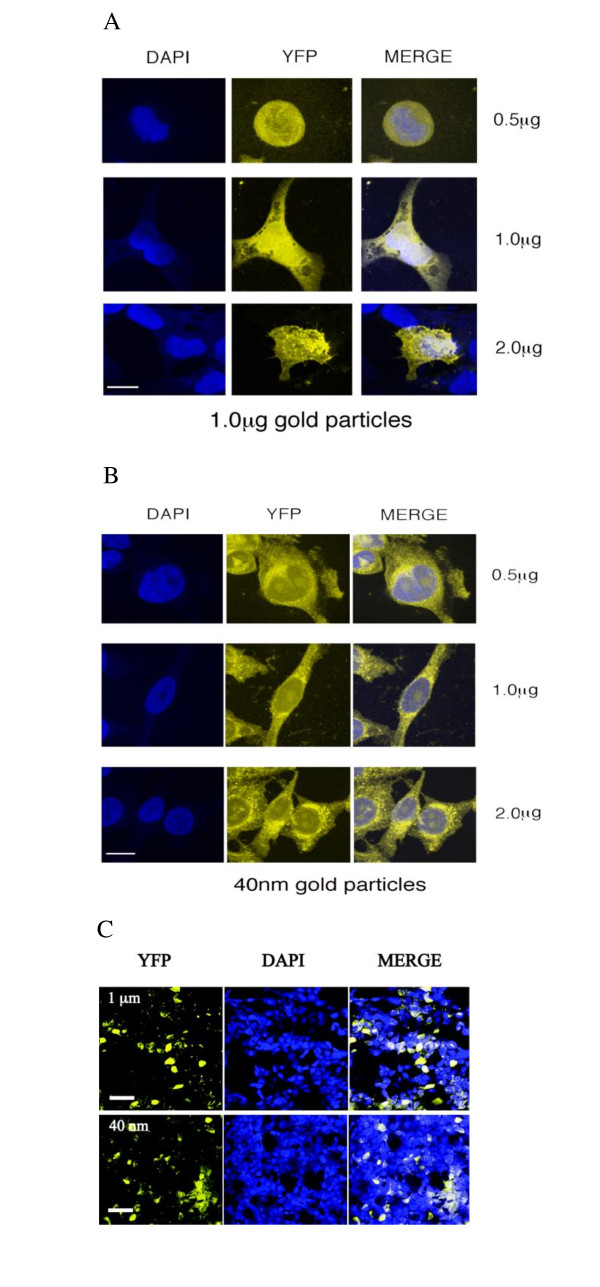
**Nano and micro-particles show similar efficiency when transfected into HEK 293 cells**. **A and B**. Cells were transfected with a single 'shot' containing 0.5, 1 or 2 μg of DNA encoding yellow fluorescent protein (YFP) precipitated onto gold projectiles. The cells were incubated for 24 h, fixed, and then examined using a confocal microscope. For further details see text. Images are representative of > 1000 cells from at least 6 transfections. Cells transfected with uncoated gold particles or those with 0.25 μg DNA resulted in no expression (data not shown). YFP = yellow fluorescent protein; DAPI = diamidinophenylindole which reveals cell nuclei; MERGE = combined images. Scale bar = 15 μm. **C**. Typical low resolution images of HEK293 cells biolistically transfected with 1 μg of YFP-DNA using 1 μm or 40 nm gold projectiles. Scale bar = 100 μm.

Transfection efficiency was monitored by examining expression of yellow fluorescent protein (YFP) in > 1000 cells in n separate transfections (examples are shown in Figure 2). Using 1 µm microparticles coated with 1 μg DNA (per shot) revealed an efficiency of 27 ± 4% (n = 12); transfections using 40 nm nanoparticles under the same conditions revealed a similar efficiency (25 ± 4%, n = 12). For both samples we observed lower transfection efficiency at 0.5 μg DNA (8.8 ± 2.7% and 7.8 ± 2.1% for micro and nano-particles respectively, n = 6) and 2 μg DNA (14.5 ± 3.6% and 12.9 ± 3.8%, n = 6), with no observable fluorescence at 0.25 μg (n = 6), indicating an optimum of 1 μg for transfection as shown in previous studies using microparticles e.g. [14]. No transfection occurred when either 1 μm or 40 nm projectiles formulated with 1 μg DNA were added directly to the cell media (n = 3; > 1000 cells examined)

### Tissue damage

**Figure 3:**
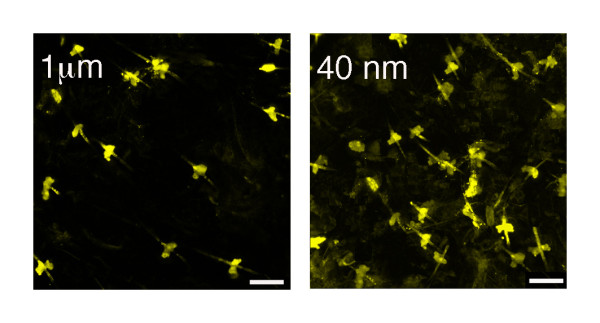
**Ear cells**. Typical images of ear cells biolistically transfected with 1 μg of YFP-DNA using 1 μm or 40 nm gold projectile**s**. These images are from a section of tissue at a depth of 20 µm. Scale bar = 10 µm. Examination of > 10 images revealed ~ 10% less YFP labelled cells in the microparticle-transfected cells, consistent with our data showing more tissue damage in these samples.

A particular problem with biolistic transfection is cell/tissue damage e.g. [8,11-15]. In cultured cells this type of transfection results in a zone of dead cells around the shot. This zone of cells is quite distinct to the cells that may have been blown away by the helium gas which is used to deliver the microparticles; for both 1 μm and 40 nm microparticles similar numbers of cells were dislodged by the gas blast. To quantify cell damage we calculated the extent of dead or damaged cells as defined using DAPI staining (which labels nuclei and shows enhanced fluorescence with dead or damaged cells) 24 h post transfection. These data revealed dead cell diameters from the centre of the shot of 63.7 ± 3.5 µm for 1 μm particles and 21.7 ± 1.3 µm for 40 nm particles (n = 6; significantly different, Students t-test, p < 0.05). Thus the use of nanoparticles results in considerably less cell damage. To further explore the problem of tissue damage, we examined 100 randomly selected cells from 6 mouse ear samples transfected with micro and nanoparticles, and examined their viability using DAPI. The data revealed that 9 ± 2% of cells were damaged in the nanoparticles-transfected samples, whereas 22 ± 3% were damaged in the microparticle-transfected tissue (significantly different, Students t-test, p < 0.05). Thus again the use of nanoparticles significantly reduced cell damage. Biolistic transfection of ear samples with 1 μm or 40 nm projectiles coated with 1 μg YFP-DNA supported these conclusions: examination of 10 randomly selected images revealed fewer YFP-labelled cells in the former when compared to the latter (Figure 3).

### Effect of spermidine and calcium chloride

**Figure 4:**
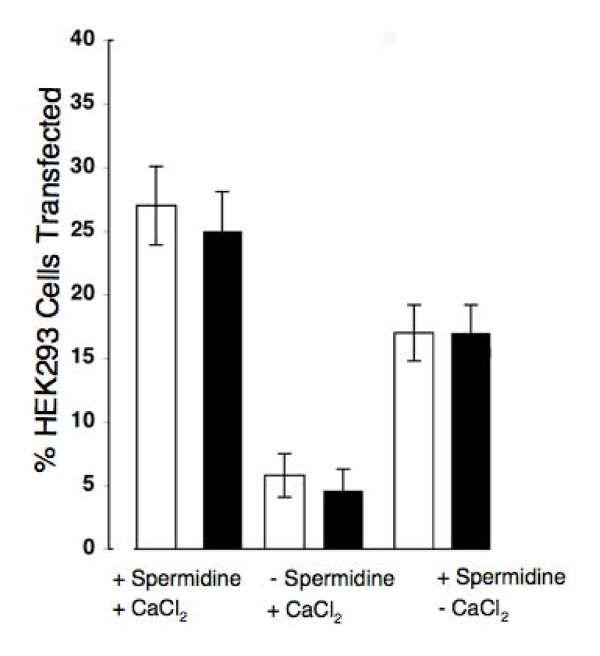
**Comparison of biolistic transfection efficiencies in HEK293 cells using micro and nano particles under different conditions**. DNA encoding YFP was routinely precipitated onto gold projectiles using spermidine and CaCl_2_. Removal of one of these components resulted in a significant loss of protein expressed, as measured by the numbers of fluorescent HEK 293 cells recorded by confocal microscopy 24 h after transfection with gold particles coated with 1 μg DNA. In the absence of spermidine, transfection efficiency was reduced to 6.1 ± 2.2 and 4.6 ± 1.7 for 1 µm and 40 nm gold particles respectively (n = 12; significantly different, Students t-test, p < 0.05), and in the absence of only CaCl_2 _transfection efficiency was reduced to 17.2 ± 3.2 and 16.6 ± 2.5 respectively (n = 12; significantly different, Students t-test, p < 0.05). Cells transfected with projectiles containing no DNA, or that had been prepared with neither spermidine nor CaCl_2_, resulted in no fluorescent cells (data not shown, n = 12).

To determine if there were differences in DNA adherence to the different projectiles, we examined the effect of removing two of the preparation reagents - spermidine and CaCl_2_- on the efficiency of transfection (Figure 4). The absence of spermidine reduced transfection efficiency to 6.1 ± 2.2 and 4.6 ± 1.7% for 1 µm and 40 nm particles respectively (not significantly different, Students t-test, p > 0.05, n = 6). The absence of CaCl_2 _had similar reductions in efficiency in the two types of microparticles although had less of a deleterious effect (17.2 ± 3.2 and 16.6 ± 2.5% respectively; not significantly different, Students t-test, p > 0.05, n = 6). There was no significant transfection when both spermidine and CaCl_2 _were removed from the preparation, or when untreated projectiles were used (data not shown). These data suggest that the adherence characteristics of DNA to the two different sized projectiles are similar.

### Depth penetration

For many experiments and for therapeutic applications it is advantageous to transport genetic material deep into living tissue. To explore if nanoparticles are as effective as microparticles, we biolistically transfected mouse ear tissue and then examined sections of tissue to determine the location of the particles. These studies revealed that there was no significant difference in the maximum depth penetration of microparticles when compared to nanoparticles: maximum depths recorded were 50 ± 11 µm and 31 ± 6 µm for micro and nano-particles respectively (n = 6 transfections for each; not significantly different, Students t-test, p > 0.05). We have previously used depth penetration to compare the effects of the modified versus the classic gene gun barrel using agar blocks, which are a considerably softer target than skin, and, using microparticles, the data revealed a maximum depth penetration of ~500 µm for the modified gene gun barrel, compared to ~100 µm for the original barrel [11]. Thus penetration through skin is ~10x less effective than through the agar blocks we used previously, although, given the potential use of the gene gun for therapeutic applications, a comparison of particle depth through skin is probably a more useful evaluation of this characteristic.

### Examination of small cellular structure

**Figure 5:**
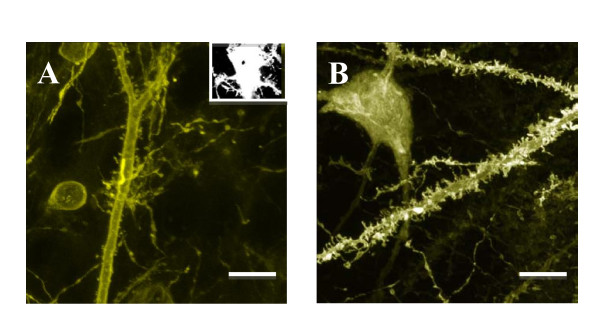
**Comparison of Purkinje cell labelling after transfection with micro or nano-particles**. Images reveal that greater structural detail is visible when brain slices are biolistically transfected with 40 nm projectiles (A) when compared to those transfected with 1 μm projectiles (B). These are typical examples representative of >10 samples, and reveal exquisite detail of the spines when transfected with the smaller particles. Scale bars = 5 μm. Inset: 1 μm gold particles can be readily visualised inside cells; this is an example of a brain slice neurone. Scale bar = 10 μm.

The small size of the particles permits them to transfect regions of cells that are not efficiently transfected with larger particles. Biolistic transfection of brain slices using 40 nm or 1 μm particles reveals YFP-labelled Purkinje cells, but the resolution of small cellular structures, such as the dendritic spines, is greatly enhanced when using 40 nm particles (Figure 5).

## Discussion

We describe here the use of nanometre-sized particles to transfect cell and tissue samples using biolistic methods. The method for preparing these particles is the same as that for the preparation of microparticles, which are widely used in biolistics. Our data show that efficiency of nanoparticles is similar to that of microparticles, and that their use results in less tissue damage. Efficiency of transfection in culture cells can be considerably higher using other transfection techniques using lipids, polymers or electroporation, for which we have observed levels > 80% in HEK 293 cells (data not shown). The particular advantage of the gene gun, however, is its use for transfecting cells that cannot be easily transfected by these methods, and for transfecting cells deep in tissues. There is currently much interest in using the gene gun for delivery of DNA vaccines into skin and muscle, and in gene therapy e.g. [8-10,19-21]. The introduction of dystrophin cDNA in a mouse Duchenne dystrophin model using microparticles, for example, has resulted in detectable levels of dystrophin protein for up to 60 days after bombardment [22]. The discovery that smaller projectiles are equally effective but cause less tissue damage could therefore have a significant impact on the feasibility of biolistic transfection as a therapeutic technique. It may also be possible to modify the nanoparticles, e.g. with polyethyleneimine to create cationic gold particles, which have been shown to deliver increased amounts of DNA, although the efficiency and damage-propensity of these particles has not been examined [23-25]. The use of such small particles as efficient carriers of genetic material also enhances the prospects of efficiently transfecting smaller organisms or specific regions of cells such as dendritic spines.

## Conclusions

Nanoparticles have similar efficiency to microparticles for biolistic transfection, and can be used to efficiently transfect small cells, organelles, or specific cell regions. In addition their smaller size results in less tissue damage, indicating that they are more appropriate for use where tissue damage is a problem.

## Authors' contributions

Both authors designed the study and performed experiments. SCRL had the major role in writing the manuscript, and JAOB had the major role in preparing the bullets and performing the biolistic transfections. All authors read and approved the final manuscript.
